# Combating Methotrexate Resistance in Cancer Treatment: A Review on Navigating Pathways and Enhancing Its Efficacy With Fat-Soluble Vitamins

**DOI:** 10.1155/sci5/8259470

**Published:** 2025-04-16

**Authors:** Muhsina Kakkadath, Disha Naidu, S. K. Kanthlal, Khan Sharun

**Affiliations:** ^1^Department of Pharmacology, Amrita School of Pharmacy, Amrita Vishwa Vidyapeetham, Kochi 682041, Kerala, India; ^2^Graduate Institute of Medicine, Yuan Ze University, Taoyuan 32003, Taiwan

**Keywords:** cancer, fat-soluble vitamins, methotrexate, resistance, transporter

## Abstract

Methotrexate (MTX), a potent analogue and antagonist of folic acid, is a first-line treatment for rheumatoid arthritis, IBD and cancer. The development of MTX resistance contributes to the reduced efficacy and development of adverse reactions, forcing clinicians to withdraw treatment early. This drawback requires combinational approaches to combat the resistance and enhance the efficacy and safety of MTX. To provide a brief overview of MTX resistance and strategies to mitigate its aftereffects in cancer therapy, a literature-based search was conducted using keywords such as cancer pathology, MTX mechanism and resistance, S100A4, folate uptake, folate efflux, P-glycoprotein, beta-catenin and anticancer properties of Vitamins A, D, E and K. Investigations encompassing in vitro studies, in vivo studies and clinical trials were reviewed to identify the mechanisms of resistance induced by MTX and the potential benefits of coadministering fat-soluble vitamins with existing anticancer drugs. Derivates of Vitamin A could target cancer stem cells and increase chemotherapy sensitivity in non–small cell lung cancer. Similarly, calcitriol and cytotoxic medications exhibit additive or synergistic effects. Existing research revealed that fat-soluble vitamins can inhibit drug transporters, such as P-glycoprotein, which inhibit drug efflux, improving chemotherapy efficacy in cancer. As personalised medicine continues to evolve, incorporating combination approaches with MTX and fat-soluble vitamins holds promise for enhancing treatment efficacy, which can counteract MTX resistance via multiple pathways and improve the safety profile.

## 1. Introduction

Methotrexate (MTX), formerly known as amethopterin, is one of the folic acid (FA) antagonists initially developed in the 1940s to treat a variety of cancers and noncancerous conditions [1]. MTX (4-amino-10-methylfolic acid) is an antagonist and analogue of FA that prevents cell division, repair and synthesis of DNA. Low-dose MTX showcases a range of beneficial properties, including anti-inflammatory, antiproliferative, antirheumatoid and immunomodulating effects, making it effective as a maintenance therapy for conditions such as Crohn's disease, vasculitis and refractory atopic dermatitis, while also serving as a first-line treatment for early rheumatoid arthritis (RA) and other inflammatory arthropathies [2, 3]. MTX is widely used to treat osteosarcomas (OSs), non-Hodgkin's lymphomas and a few other cancers [4–6].

MTX disrupts the synthesis of tetrahydrofolate (THF), an essential cofactor vital for synthesising nucleotides, by blocking the action of the enzyme dihydrofolate reductase (DHFR). This scarcity of THF impedes the synthesis of DNA and RNA, causing cell death [7]. As weekly maintenance chemotherapy, MTX is given at doses ranging from 12 mg intrathecally to 20 mg/m^2^ orally, intramuscularly or intravenously for acute lymphoblastic leukaemia (ALL) to intravenous dosages up to 33,000 mg/m^2^ for some additional conditions. High-dose MTX (HD-MTX) is defined as an intravenous dose of 500 mg/m^2^ or more used to treat a range of adult and paediatric malignancies, including OS, lymphoma and ALL [8, 9]. The extended exposure to high concentrations of MTX that results from delayed renal excretion makes HD-MTX a medical emergency since it can induce severe and perhaps fatal toxicity. Leucovorin rescue and sufficient supportive care are needed [10]. Still, it is a commonly used anticancer drug and is the focus of more than a thousand active clinical research. Because of its high toxicity, it is frequently stopped too soon, which lowers its potential efficacy [7].

MTX, despite its cost-effectiveness and efficacy, poses potential toxicity concerns leading to drug withdrawal, drawing significant attention due to adverse events. Its side effects fall into two categories: symptomatic, rarely fatal and those initially asymptomatic but potentially fatal, warranting close medical monitoring [1, 11]. However, its use comes with notable toxicity concerns, prompting the need for careful monitoring and sometimes leading to drug withdrawal. Given these concerns, researchers and clinicians have explored various strategies to mitigate MTX-related adverse events while maintaining its therapeutic benefits. One approach involves combination therapy, where MTX is used in conjunction with other supplementations to achieve disease control with lower MTX doses, thereby potentially reducing its toxicity. This review explores the complex landscape of MTX resistance, including pathways such as reduced folylpolyglutamate synthetase (FPGS) activity leading to defective polyglutamation, dysregulation of DHFR, thymidylate synthase (TYMS), S100A4 and gamma-glutamyl hydrolase (GGH), and increased antifolate efflux owing to ATP-driven MDR efflux transporter overexpression. We also discussed approaches to address MTX-resistance-related adverse events, focussing on combination therapies with fat-soluble vitamins and enhancing therapeutic outcomes.

## 2. Journey of MTX

In the 1940s, Yella Pragada Subbarow and his team achieved a milestone by isolating FA from liver tissue and synthesising it from microbial sources in 1945. Simultaneously, Boston's Children's Hospital researchers suggested using a FA antagonist to treat childhood leukaemia. Inspired by this, Subbarow synthesised aminopterin and its more stable analogue, amethopterin. In 1948, amethopterin was successfully used to treat childhood leukaemia, eventually becoming the groundbreaking drug known as MTX, heralding a transformative moment in medical history [12, 13].

American laboratories discovered that cortisone and aminopterin inhibited the proliferation of mesenchymal cells in the 1950s. In 1951, aminopterin's antianabolic, anti-inflammatory and antiproliferative effects in connective tissue were demonstrated by Gubner and associated to be similar to those of cortisone. This finding provided the impetus for a 1951 investigation into the potential uses of the results in the management of RA [13]. Phillip Hench and colleagues received the Nobel Prize in 1950 for their research on the use of corticosteroids in treating RA. The rheumatology community at the time was largely focused on corticosteroids, and MTX was not widely explored due to the enthusiasm surrounding corticosteroids and reservations regarding the use of an anticancer medication for a ‘benign disease' like RA. Not until 1972, when practicing rheumatologist Rex Hoffmeister reported notable benefit from intramuscular MTX administered at weekly doses of 10–15 mg in 29 RA patients—11 of whom saw substantial clinical improvements and 14 of whom saw moderate improvements over a 25-month course of treatment—did Hoffmeister's research. However, when the MTX dose was reduced to less than 10 mg per week or discontinued, more than 80% of these patients experienced arthritis flares [14].

### 2.1. How It Works

#### 2.1.1. MTX and Folates

Folates are essential for one-carbon metabolism, which is necessary for synthesising purines, thymidylate and DNA replication. Nearly 60 years ago, the antifolate MTX was logically developed to temporarily remit cancer by potently blocking the folate-dependent enzyme DHFR [15]. In the presence of coenzyme NADPH, DHFR aids the reduction of 7,8-dihydrofolate (DHF) to 5,6,7,8-THF: DHF + NADPH + H^+^ ⟶ THF + NADP^+^. Maintaining intracellular stores of THF and its derivatives, essential cofactors in one-carbon metabolism, depends on this enzyme activity [16]. The RFC1, which is encoded by SLC19A1 and found in the cell membranes of cancerous and foetal cells, is the main route by which MTX penetrates cells. FPGS catalyses the polyglutamation of MTX inside the cytosol, which improves its activity and relies on both time and concentration. Since generic folate diffusion is limited, the specialised transporter RFC1 is essential for mediating the entry of MTX into the cell and facilitating folate transfer [17, 18].

MTX and its polyglutamate inhibit DHFR, which stops dihydrofolate from converting to THF, disrupting de novo pyrimidine and purine synthesis. Due to this interference, cellular quantities of 10-formyl-THF, which is essential for purine synthesis, are decreased, and 2-deoxyuridine-5-monophosphate (dUMP) is methylated to 2-deoxythymidine-5-monophosphate (dTMP). The inhibition of purine synthesis is further aided by MTX's direct effect on 5-aminoimidazole 4-carboxamide ribonucleoside (AICAR) transformylase and glycinamide ribonucleotide (GAR) transaminase. Longer chains of MTX polyglutamates than three glutamate residues are not exportable from the cell and are hence deglutinated by the lysosomal *γ*-glutamyl hydrolase. Intracellular MTX levels stabilise when polyglutamation and deglutamation are balanced. Finally, the ATP-binding cassette (ABC) proteins (ABCC1-4 and ABCG2) export MTX from the cell [19].

MTX can cause vitamin B12 and folate deficits because it acts as a folate antagonistic property, disrupting normal metabolic pathways required for DNA synthesis and RBC. Patients under MTX therapy, especially those with RA, are more likely to develop these deficiencies, which may result in increased homocysteine levels and complications such as macrocytic anaemia and neurological disorders. Clinical investigations have indicated that even with concurrent FA administration, individuals on MTX may still develop vitamin B12 deficiency, prompting more research to determine the necessity for routine monitoring and further supplementation regimens to effectively compensate for these risks [20].

However, MTX is ineffective in myeloid leukaemia (AML) because leukaemic cells are unable to polyglutamate the drug. Compared to ALL, leukaemic blasts in the majority of AML subtypes have lower levels of FPGS and higher levels of gamma-glutamyl hydrolase (GGH), which together diminish the production and stability of long-chain MTX polyglutamates (MTX-PG). Since nonpolyglutamated MTX is quickly exported from cells, such deficiencies make MTX ineffectual in the majority of AML cases. On the other hand, M5-AML and some late-relapsed M7-AML subtypes exhibit sensitivity to MTX and effective MTX polyglutamylation [21].

Despite its efficacy in multiple indications, MTX faces limitations in broader applications owing to its higher resistance rates.

#### 2.1.2. MTX and Cancer [22]

MTX is widely used in oncology due to its strong antimetabolite characteristics, specifically targeting rapidly growing cancer cells. It is a cornerstone in the treatment of ALL, especially in paediatric and adult patients, and is beneficial in preventing cancer progression [23]. MTX is also used to treat OS, and it is given in high dosages after surgery to eliminate any surviving tumour cells. For primary central nervous system lymphoma (PCNL) and leptomeningeal metastases, MTX is given intrathecally or intravenously resulting in high CNS concentrations, showing excellent efficacy. Furthermore, MTX has been effective in combination regimens for non-Hodgkin lymphoma (NHL), improving treatment results.

Although it is effective in other solid tumours such as breast cancer, lung cancer, and head and neck cancers, as well as haematologic malignancies, factors such as resistance mechanisms, pharmacokinetics, toxicity profiles and therapeutic drug monitoring (TDM) standards vary widely depending on the tumour type and individual patient characteristics [22, 24–26].

## 3. MTX Resistance

Neoplastic cells may be innately resistant or acquire resistance to MTX. The term ‘MTX resistance' describes how cells or organisms become less effective or responsive. MTX's efficacy in treating certain conditions may be diminished over time as cells evolve defence mechanisms against or escape its effects. The resistant pathways are as follows:

• Reduced absorption of antifolate as a result of RFC and proton-coupled folate transporter (PCFT) dysfunction.
• Elevated antifolate efflux brought on by ATP-driven MDR efflux transporter overexpression.
• DHFR, TYMS overexpression and its mutation that reduces its affinity for antifolates (MTX).
• S100A4 overexpression reduces protein's affinity for antifolates.
• Inactivating mutations or reduced FPGS expression resulting in ineffective antifolate polyglutamation.
• Elevated expression of GGH [15, 27–31].
• Decreased TYMS activity or affinity for folate antagonists.

### 3.1. Impaired Antifolate Uptake due to Loss of RFC and PCFT Function

Human tissues and tumours contain three main folate uptake systems, namely,

a. PCFT
b. Folate receptors (FRs)
c. RFC [32].

**Figure 1:**
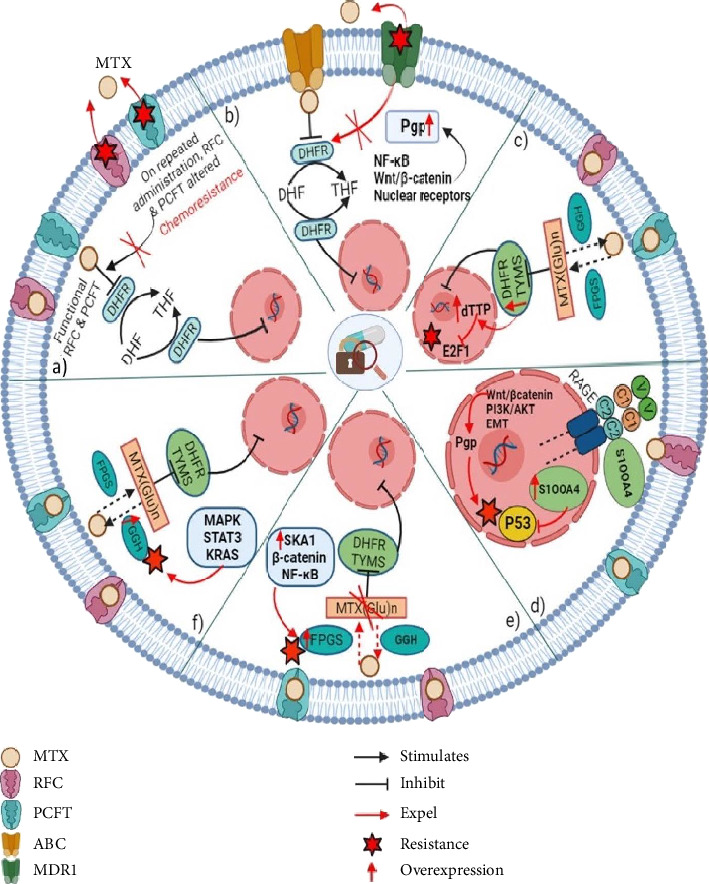
Major mechanism contributing MTX resistance in cancer cells. Showing the multiple pathways of methotrexate resistance, such as (a) impaired antifolate uptake due to the loss of RFC and PCFT function. (b) Increased antifolate efflux due to the overexpression of ATP-driven MDR efflux transporters. (c) Overexpression of DHFR, TYMS and its mutation that decreases its affinity for antifolates. (d) Overexpression of S100A4, which decreases its affinity for antifolates. (e) Defective antifolate polyglutamation due to decreased FPGS expression and/or inactivating mutations. (f) Increased expression of gamma-glutamyl hydrolase.

MTX is transported into cells with the help of the human RFC (or SLC19A1), which acts as the main folate importer. Due to its extensive expression in various cell types and tissues, hRFC is the primary folate transporter in mammals. It is essential for the effective distribution of folates throughout the body. Furthermore, depletion and genetic variations of hRFC lead to drug resistance [18, 33–35]. MTX resistance results from reduced expression and mutations in the RFC gene, which hinder tumour cells' ability to absorb antifolates effectively. On the other hand, some mutations in the RFC gene may increase the affinity for folate, which could result in higher intracellular folate concentrations, competition with MTX and decreased therapeutic effectiveness. [36]. The multiple pathways of MTX resistance are depicted in Figure 1.

Hereditary deficits in folate absorption are linked to mutations in PCFT. Lower expression scores of PCFT, RFC and FR in colorectal cancer and unique DNA methylation patterns indicate complex and possibly type-specific regulation of folate transporter expression, highlighting the role of epigenetic mechanisms in modulating these patterns [33]. A meta-analysis on the association between cancer risk and the RFC1 G80A polymorphism was carried out by Huang S. The findings demonstrated a strong correlation, especially in some ethnic groups, between the RFC1 G80A polymorphism and the incidence of solid cancer [37]. PCFT activity in the MTX-resistant Hela R1 cell line is associated with hypermethylation, which suggests that methylation levels in the GC-rich PCFT promoter may control the transporter. A mechanism for enhanced intestinal folate absorption under specific circumstances is suggested by the hypomethylation seen during folate deprivation and may stimulate PCFT expression [38]. The investigation of antifolate resistance in eight different myeloma cell lines showed that resistance and the increase of DHFR protein in response to PDX are correlated. Furthermore, the expression and function of RFC were linked to the sensitivity to PDX in these cell lines, underscoring the importance of these pharmacologic and genetic factors in the antifolate response [29].

### 3.2. Increased Antifolate Efflux due to Overexpression MDR Efflux Transporters

The term multidrug resistance (MDR) refers to the development of drug resistance that can eventually lead to resistance to several different medications that are structurally and functionally unique from the initial drug [39]. The fundamental causes of MDR have recently come to daylight. These include the disruption of various cell signalling pathways involved in cell apoptosis, enhancement of tumour cell proliferation and DNA damage repair capacity, induction of autophagy in tumour cells, modification of the microenvironment of tumour cells, aberrant epigenetic regulation, rise in cancer stem cells, drug target mutation and low intracellular active-drug concentration [40].

Chemotherapy failure is attributed to a reduction in intracellular active medicines, which is caused by an increase in drug efflux and a decrease in drug influx due to drug transporters. Among these, the most significant feature is the rise in drug efflux brought on by the overexpression of the ABC superfamily-binding cassette in cancer cells [41]. Numerous medications and xenobiotics are actively pumped through the cellular membrane by ABC transporters. ATP-binding domains, sometimes referred to as nucleotide-binding domains (NBDs), and transmembrane domains are the two fundamental components of ABC transporters.

SLC transporters, such as OAT1, OAT3, OAT4, OATP1A2 and OAT1B3, and ABC transporters, such as MRP2, MRP3, MRP4, MRP5 and BCRP, are responsible for facilitating the transportation of MTX [42]. MDR-associated proteins (MRPs/ABCCs), P-glycoprotein (P-GP/ABCB1) and breast cancer resistance protein (BCRP/ABCG2) are among the at least 11 ABC transporters that have been linked to the development of MDR [43]. The alteration of ABC transporters was connected with nuclear receptors [44] and post-translational modification as well as cell signalling pathways like Wnt/β-catenin [45]. NF-κB signalling-mediated P-GP overexpression is one of the causes of MDR [46]. Chemoresistance in juvenile ALL is defined by the increased expression of drug efflux transporters such as BCRP and MRP4, which lowers MTX intracellular retention. High MRP4 expression is associated with ex vivo MTX resistance, and elevated MRP4 and BCRP expression is linked to less MTX-polyglutamate buildup, indicating their significant role in decreased MTX responsiveness and shorter overall survival in childhood ALL [47].

### 3.3. Overexpression of DHFR, TYMS and Its Mutation

By specifically targeting the enzymes, TYMS and/or DHFR, this medication prevents the production of DNA [48]. However, MTX resistance hinders its therapeutic use in treating cancer [49]. The overexpression of DHFR and TYMS, and reduction in MTX poly-γ-glutamylation are the mechanisms of MTX resistance in malignancies [31, 50]. RFC1 downregulation and increased expression of DHFR because of gene amplification were identified as potential resistance mechanisms in H9-12 and H9-200 cells, according to the expression analysis of the proteins known to be the significant determinants of antifolate pharmacology [51]. Recent studies reveal that the mutation in the DHFR gene as well as TYMS is responsible for the decreased uptake of MTX [50, 52].

Wangh et al. demonstrated that metformin effectively hinders cell cycle progression, curtails carcinogenesis, suppresses DHFR activity and disrupts nucleotide metabolism, collectively heightening the sensitivity of hepatocarcinoma cells to MTX. Mechanistically, metformin supports the lysosomal degradation of DHFR protein and transcriptionally represses DHFR via E2F4. Interestingly, metformin enhances the hepatocarcinoma organoid responsiveness to MTX without harming organoids produced from normal liver tissue, highlighting DHFR as a possible target for therapy to overcome hepatocarcinoma resistance [53]. MTX-resistant HT29 cells with double minutes (DMs) exhibited high MSH3 expression, which resulted in elevated ecDNA levels, DHFR amplification and drug resistance. The reduction of ecDNAs, HSRs and DHFR expression upon MSH3 depletion underscores the significance of MSH3 in regulating cDNAs via DNA double-strand break repair and its bearing on drug resistance in cancer [54]. MTX resistance in melanoma cells is caused by DHF depletion, E2F1 activation and subsequent upregulation of DHFR and TYMS, which encourages the accumulation of dTTP. Excess dTTP inhibits E2F1-mediated apoptosis, which contributes to MTX resistance. This results in DNA single-strand breaks, Chk1 activation, cell cycle arrest in the S phase and protection from apoptosis [16].

Kim et al. explored the DHFR gene amplification in MTX-resistant colon cancer and ALL. The results showed a tandemly amplified area covering 11 genes with chromosomal abnormalities. Mutations in the MSH and MLH genes, such as inversions and frameshift insertions, point to possible causes of chromosomal breakage and dysregulation of mismatch repair, providing information about the mechanisms underlying genomic rearrangements and the evolution of drug resistance [55]. In Leishmania, a chemogenomic screen using MTX selection produced 20 mutants with reduced MTX sensitivity. This screen also revealed surprising mutations in ALO and methyltransferase LmjF.17.1130, along with unique point mutations in genes associated with MTX resistance (FT1, DHFR-TYMS, PTR1). Although FT1 and DHFR-TYMS mutations showed phenotypic significance in MTX resistance, the resistance they imparted was reversed by overexpressing wild-type ALO and LmjF.17.1130, indicating different mechanisms underpinning antifolate resistance in Leishmania [55, 56].

### 3.4. Overexpression of S100A4

The S100 protein family includes its significant member S100A4, which has two calcium-binding motifs. Its main function is to encourage the spread and metastasis of cancer. In addition to its effects on cell motility, invasion and autophagy, S100A4 has been linked to fibrogenic and inflammatory processes. In addition to being expressed in tumours, upregulated S100A4 levels are linked to several pathophysiological processes that do not involve tumours [57–60]. Recent studies show that S100A4 promotes tumour metastasis [61–63]. Huo et al. demonstrate that S100A4 encourages the growth of lung tumours by blocking autophagy in a way that is dependent on *β*-catenin signalling and the S100A4 and receptor for advanced glycation end products (RAGE) interaction [58]. Additionally, S100A4 plays a crucial function in chemoresistance; multiple studies demonstrate that overexpressing S100A4 regulates chemotherapy resistance [64, 65].

The S100A4 controls several signalling pathways, including PI3K/AKT and Wnt/β-catenin, which are directly linked to invasion, migration, epithelial-mesenchymal transition (EMT) and resistance in cancer cells [66, 67]. S100A4 deactivates p53, prevents it from being phosphorylated and affects p53's ability to reduce tumours [68, 69]. Additionally, they control the activity of Matrix Metallopeptidase-2 (MMP-2), MMP-9, MMP-11 and plasmin, which impacts the disintegration of the extracellular matrix, a crucial requirement for tumour invasion and metastasis [70, 71]. Furthermore, S100 proteins exhibit a strong affinity for calcium ions, which they use to modulate cytosolic Ca2+ levels, contributing to apoptosis, cell cycle dysregulation and elevated expression of the Multidrug Resistance Gene-1 (MDR1) [72, 73].

In multiple MTX-resistant cells, S100A4 is increased. The HT29 colon cancer cells' susceptibility to MTX is decreased by the overexpression of S100A4, but chemosensitivity is increased by its suppression. This indicates that S100A4 is essential for MTX resistance. S100A4 mRNA expression is doubled in HT29-sensitive cells upon *β*-catenin transfection, indicating that the Wnt/β-catenin pathway is involved in mediating S100A4 transcription. P-GP, another target of the Wnt signalling pathway linked to MTX resistance, is remarkable since it broadens our knowledge of these pathways [27]. However, it has been observed that TRIM37-induced chemoresistance is partially reliant on the Wnt/β-catenin signalling pathway being activated [74].

### 3.5. Defective Antifolate Polyglutamation

An FPGS substrate, MTX, must be polyglutamylated to effectively retain intracellularly and target tumour cells specifically [75]. MTX polyglutamation involves adding two to seven glutamic acid residues, which are catalysed by FPGS and reversed by GGH. FPGS enhances the intracellular retention of folates by catalysing a rate-limiting polyglutamylation step in the FA metabolic cycle [76–78]. Yu et al. employed the whole-exome sequencing analysis in relapsed childhood ALL and discovered relapse-specific mutations in the FPGS gene in one patient. In these cases, FPGS mutations probably contributed to treatment resistance and relapse because they directly resulted in lower enzymatic activity, which markedly reduced MTX polyglutamation [79]. MTX-induced FPGS deficiency arises through transcriptional downregulation or the acquisition of inactivating mutations, according to studies conducted on cancer cells and cell lines taken from patients who have relapsed [80, 81].

In OSOS, high SKA1 levels are linked to de novo resistance to the drug MTX and worse patient outcomes. SKA1 overexpression blocks MTX activation by lowering FPGS levels. Silencing SKA1 restores MTX sensitivity in resistant cells, making it a potential target for overcoming resistance and improving survival [82]. Another study by Liu et al. demonstrates that *β*-catenin promotes leukaemia cells resistant to MTX by inhibiting FPGS expression via NF-κB. The results obtained suggest that the *β*-catenin-NF-κB-FPGS pathway may be linked to MTX resistance, suggesting that *β*-catenin is a good target for combination therapies in the management of ALL [83].

### 3.6. Increased Expression of GGH

GGH is a key element in antifolate pharmacology and folate metabolism since it dynamically controls its activity in cancer cells. To maintain the metabolic equilibrium of folates and the therapeutic efficacy of antifolates, GGH catalyses the removal of gamma-polyglutamate tails from folylpoly-/antifolylpoly-c-glutamates, guaranteeing their successful export from the cell [84]. In cancer, the overexpression of GGH has been observed [85, 86]. MTX and other antifolate medications cause cell resistance, which correlates with high GGH expression. The dysregulation of several cancer-related gene pathways, such as those governing cell cycle, cell motility, MAPK, STAT3, KRAS signalling and immunological responses, may be linked to the overexpression of GGH. Furthermore, by changing the nature of immune cell infiltration, GGH overexpression may lessen the immunological response to tumours [87]. Using MTX-resistant lymphoma cell lines, Hayano et al. examined gene expression and discovered that GGH was highly expressed [88]. It was shown that overexpressing S-Phase Kinase–Associated Protein 2 (skp2) in MTX-resistant OS cells enhanced their invasive, migratory and adhesion capabilities as well as the EMT. Tumour cells were more sensitive to MTX when Skp2 was inhibited with shRNA [89].

### 3.7. Decreased Thymidylate Synthase Activity

Impaired TYMS activity or altered affinity for antifolate drugs such as MTX contributes greatly to tumour cell resistance to these treatments. Since TYMS is a key enzyme in the de novo synthesis of thymidine and is necessary for DNA replication and repair, antifolate medications such as MTX target it. Increased TYMS expression, which restores thymidine synthesis despite pharmacological suppression, or decreased intracellular drug accumulation are common resistance mechanisms. Studies have revealed that the overexpression of TYMS in malignant pleural mesothelioma cells leads to pemetrexed resistance, with larger levels of deoxythymidine monophosphate (dTMP) found in resistant cells [90].

Hence, TYMS-targeted therapy may foster translational autoregulation, enhancing TYMS levels by two to four times, enabling tumour cells to avoid harmful effects [90, 91]. Resistance may arise from reduced cellular uptake or increased efflux of antifolate drugs, as demonstrated in ovarian and colon cancer cells treated with a novel TYMS dimer disruptor [92]. Furthermore, strategies like antisense oligodeoxynucleotides targeting TYMS mRNA have shown promise in preclinical investigations to mitigate overexpression-mediated antifolate resistance.

Together, these resistance pathways cause MTX's intracellular concentrations and efficacy to drop, which limits the drug's capacity to obstruct folate-dependent processes and stop the proliferation of cancer cells. Comprehending these pathways is essential to formulating tactics to surmount or circumvent the resistance and augment the therapeutic potential of MTX in the management of cancer. Hence, a comprehensive and sophisticated approach is necessary to optimise the therapeutic benefits of MTX. A targeted approach is necessary to target various resistance pathways, highlighting the necessity of professional medical advice to guarantee safe and individualised treatment plans.

Although fat-soluble Vitamins A, D and E are vital for overall health, K shows a limited direct impact on MTX resistance mechanisms. Current strategies prioritise customised combination treatments involving inhibitors, diverse modalities and coadministration of MTX with FA supplements, targeting multiple resistance pathways. Professional medical guidance is crucial for safe and personalised treatment plans.

## 4. Role of Vitamins

### 4.1. Vitamin A

Vitamin A belongs to a class of significant fat-soluble compounds that are found in both plant and animal sources (Table 1). It has an unsaturated isoprenoid chain structure and can be received through diet in two different forms: as Vitamin A (retinol and close relatives) from animal sources or as Provitamin A (carotenoids) from plants [97]. Pharmacological doses of Vitamin A have been shown to reduce the occurrence of experimentally induced tumours, correlating with epidemiological findings suggesting a link between lower cancer development and increased dietary intake of Vitamin A [98].

The two primary active metabolites of Vitamin A are retinal and retinoic acid (RA) [99]. A vitamin A derivative, all-trans RA (ATRA), regulates cell proliferation, differentiation and apoptosis and is therefore essential in cancer treatment. The development of RA derivatives and nutritional control, along with the wider anticancer properties of vitamin A that go beyond its interaction with RA receptors, offer promising options for cancer prevention and treatment [100–102]. MacDonagh et al. reported that ATRA and retinol, two vitamin A derivatives, can eradicate cancer stem cells and increase the sensitivity of non–small cell lung cancer (NSCLC) cells to the common chemotherapy medication cisplatin. This shows that vitamin A may be able to help treat NSCLC patients who are resistant to cisplatin [103]. Like this, Vitamin A may increase the sensitivity of the chemotherapeutic drug MTX in any of its resistance pathways. In another study conducted by Yudi Mulyana and team to analyse the changes in *β*-hCG levels in low-risk gestational trophoblastic neoplasia (GTN) patients following Vitamin A administration. Oral 6000 IU of Vitamin A significantly lowered *β*-hCG levels in the neoplasia patients undergoing MTX treatment. The incidence of MTX resistance was lower in the Vit-A receiving group than in the control group. Also, combinational treatment enhances greater malignancy death than MTX alone. This implies that Vitamin A daily may increase the efficiency of MTX in lowering *β*-hCG levels and maybe decrease the occurrence of chemoresistance [95]. Vitamin A also enhanced the efficacy and diminished the resistance of other commonly used chemotherapeutic agents as well. In advanced cervical carcinoma treatment, Vitamin A supplementation (8000 IU/8 h) with neoadjuvant chemotherapy (64 weeks) in 15 patients (out of 30), enhances the therapeutic outcome of the treatment cisplatin and paclitaxel [104].

Retinoids, derived from Vitamin A, can block ABCG2 and P-GP, drug-expelling efflux transporters, from doing their work. Retinoids, that is, retinol, retinyl acetate and 13-cis RA, prevent the stiffening of cell membranes by interacting with transporters both directly and indirectly [105]. To address chemoresistance and poor prognosis in cancer treatment, Liu et al. developed a delivery system using FA–modified chitosan (CSO)–derived polymer (FA-CSOSA) to deliver doxorubicin and ATRA to tumour cells. This system combines doxorubicin and ATRA to target Pin1, a cancer growth promoter [106]. It has been demonstrated that combined ATRA and imatinib (IM) therapy effectively reduce the expression of BCR-ABL and ABCB1 genes, possibly through the differentiation of blast cells. This suggests that the therapy may be useful for patients who experience a blast crisis during their illness or who become resistant to current CML treatments [107]. Noack et al. found that ATRA shields acute promyelocytic leukaemia (APL) cells from some histone deacetylases (HDACs)'s cytotoxic effects. The ability of HDAC inhibitors (HDACis) to destroy APL cells is determined by their affinity for Class I HDACs. ATRA also lowers the expression of TYMS, which enhances its cytoprotective properties [108]. Jie et al. demonstrated a crucial role for RA in promoting IL-22 production and tempering dendritic cell function through downregulating S100 family proteins during viral hepatitis [109].

### 4.2. Vitamin D

Vitamin D originates from the sun or diet and undergoes two metabolic processes to become the powerful hormone calcitriol. Once calcitriol binds to the Vitamin D receptor (VDR), it can control various physiological processes. [110]. The importance of Vitamin D for overall health appears significant [111]. Vitamin D (1,25(OH)2D3, calcitriol) has a basic function in bone remodelling by controlling the metabolism of calcium and phosphate via its interaction with the VDR [112, 113]. In internal medicine, Vitamin D deficiency is a major issue [114]. For healthy people, the European Food Safety Authority suggests a daily dose of 600 IU (15 mg), with a maximum recommended intake of 4000 IU (100 mg). A recent recommendation by the UK Scientific Advisory Committee on Nutrition states that individuals should consume 400 IU (10 mg) of Vitamin D daily [115]. However, a wealth of studies conducted in recent years has indicated that inadequate exposure to sunshine and insufficient levels of Vitamin D may also be linked to a higher risk of numerous other nonskeletal illness, including cancer [110, 116].

Evidence from strengthened case–control studies shows inverse correlations between serum 25-Hydroxyvitamin D and 12 types of cancer, and ecological studies show lower cancer risk associated with solar radiation indices. Achieving 80 ng/mL of 25(OH)D instead of 10 ng/mL is suggested to reduce cancer incidence rates by 70 ± 10%. Experiments also show that Vitamin D inhibits tumour cell proliferation, dedifferentiation and invasion in addition to influencing stromal cells, which supports its general protective role against different types of cancers [117, 118]. Fleet et al. well acknowledged that low serum 25-Hydroxyvitamin D levels are associated with a higher risk of cancer although in an uneven and population-based manner. 1,25-Dihydroxyvitamin D is a physiologically active metabolite that controls gene transcription by binding to the VDR. This review digs into the molecular processes that underlie Vitamin D's protective effects on cancer prevention and treatment, highlighting valid mechanisms such as growth arrest and death in tumour cells triggered by Vitamin D. Other chemoprotective processes are taken into consideration, such as improved DNA repair, antioxidant defence and immunomodulation. Furthermore, the modulation of stromal cells, endothelial cells and immune system cells by 1,25-Dihydroxyvitamin D adds to its protective effect against cancer [119].

Chen and his colleagues reviewed and found that combining calcitriol with cytotoxic medicines makes sense because preclinical research shows how different medications might have additive or synergistic effects. Prostate cancer is one notable instance where Vitamin D signalling is thought to be important. This makes it a potential target for using vitamin D medicines alone or with other antineoplastic drugs to treat existing tumours [120]. A recent study reported that activating the VDR and reducing Wnt/β-catenin signalling on calcitriol treatment to MCF-7 cells resulted in G0/G1 phase arrest, inhibition of proliferation, promotion of apoptosis and DNA damage. Increased sensitivity to tamoxifen was the outcome, especially in MCF-7 stem cells. This suggests that blocking Wnt/β-catenin signalling or targeting VDR expression in breast cancer stem cells may be a promising clinical strategy for treating breast malignancies [121].

Zheng and team discovered that Vitamin D can partially overcome cisplatin resistance in oral cancer cells by acting as a sensitiser for cisplatin. This effect was linked to the modulation of Lipocalin 2 (LCN2) expression, indicating a potential way to enhance cisplatin chemotherapy in oral squamous cell carcinoma. Specifically, cisplatin increased LCN2 expression through promoter demethylation, while Vitamin D inhibited LCN2 expression through enhanced methylation [121–124].

Since Vitamin D is involved in many drug-resistant pathways in different types of cancers, its participation in MTX resistance pathways suggests that it may have a similar modulatory effect in this situation. Alam et al. reported that calcitriol administration boosted [3H]-MTX absorption by 30%–40% in hCMEC cells. Congruent increases in RFC mRNA, protein expression and functional activity in hCMEC/D3 cells and isolated mouse brain capillaries support this theory that modulating RFC function through VDR activation may be a useful tactic for improving folate uptake into the central nervous system. This is especially advantageous when folate delivery to the cerebrospinal fluid (CSF) is mediated by the choroid plexus is impaired [125, 126]. MRP1-overexpressing cells are selectively cytotoxically affected by calcitriol and its analogue calcipotriol, which limits P-gp, MRP1 and BCRP transport activities. Via high throughput screening, these vitamin D3 analogues exhibited strong inhibitory effects on MRP1-mediated doxorubicin and calcein efflux. This demonstrates their potential value in addressing tumours exhibiting an MDR phenotype because of MRP1 overexpression, supporting the sequential administration of calcitriol and additional anticancer drugs to treat MRP1-mediated MDR in clinical chemotherapy [123].

Various clinical studies have shown that Vitamin D enhances the efficacy of chemotherapy drugs by modulating the immune system and improving the sensitivity of cancer cells to these drugs. It also helps in reducing the side effects of chemotherapy, leading to better patient outcomes. Vitamin D supplementation with sorafenib improves treatment outcomes in patients with hepatocellular carcinoma, potentially enhancing the overall effectiveness of the treatment. Milkzareck et al. reported that the vitamin D analogue, tacalcitol (PRI-2191), directly activates VDR, which results in CDKN1A expression and TYMS downregulation, hence augmenting the 5-FU anticancer activity in CRC cells. Tacalcitol has two distinct actions: it affects the mechanism of 5-FU and prevents CRC cells from developing resistance [127]. Recent studies state that Vitamin D3 compounds showed a diverse effect on breast cancer through upregulating genes linked to a basal-like phenotype and negative regulators of breast carcinogenesis and downregulating genes linked to invasion, metastasis, EMT and BC stem-like cells. S100A4 gene expression decreased by 29% in response to 1*α*,25(OH)2D3. Additionally, through the downregulation of HRC, Vitamin D inhibited the development, migration and proliferation of lung cancer tumours [128–130].

A study reported that Vitamin D deficiency in paediatric patients on HD-MTX therapy was a serious concern. According to research, taking Vitamin D along with MTX may improve treatment results by lowering adverse effects and increasing the general effectiveness of treatment plans. In particular, Vitamin D may affect MTX's pharmacokinetics and pharmacodynamics, thus enabling the administration of lower doses of the medication without sacrificing its efficacy. In diseases like psoriasis and RA, when both medications are commonly used, this combination therapy may be very helpful. To maximise care and enhance patient outcomes, Vitamin D supplementation may be a viable approach to treatment regimens for paediatric patients on HD-MTX [131].

### 4.3. Vitamin E

Vitamin E encompasses four tocopherols (*α*-, *β*-, *γ*- and *δ*-tocopherols) and four tocotrienols (*α*-, *β*-, *γ*- and *δ*-tocotrienols) [132] found in a variety of foods. Although these forms are all distinctive and cannot be changed into one another, they all have antioxidant qualities. Remarkably, humans require *α*-tocopherol to meet their critical Vitamin E needs [133–135]. Vitamin E is a crucial nutrient that affects critical molecular and cellular processes and the regulation of gene expression, all of which are crucial to preventing cancer and CVD [136, 137]. Also, Vitamin E supplementation reduces the rate at which Alzheimer's disease progresses, improves working memory in adults and shields elderly patients from respiratory infections [138, 139]. Although Vitamin E is frequently used to prevent cancer [140], it is important to recognise that it also plays a role in treatment resistance. Coadministration of Vitamin E appears promising in reducing the hepatotoxic effects of MTX, even if it may be involved in resistance mechanisms. This will help patients maintain an effective therapeutic dose [141, 142]. The synergistic effect of Vitamin E and chemotherapeutic treatment reduced the viability of cancer cells and resistant cell lines (Table 2) [148, 149].

D-α-tocopheryl poly(ethylene glycol) 1000 succinate (TPGS), which the FDA has demonstrated to be a safe pharmacological adjuvant, has been reported to be able to block P-gp and reverse MDR [150]. According to current research, the star-shaped polymers d-α-TPGS and *β*-cyclodextrin (*β*-CD) are promising drug carriers to overcome MDR in the treatment of cancer [142]. Vitamin E derivatives, such as TPGS 1K and TPGS 2K, were employed by Tuguntaev R G et al. to create a nanomicellar drug delivery system for doxorubicin. The combination of P-gp inhibition and *α*-TOS-induced mitochondrial impairment resulted in steady pH-dependent drug release and significantly increased cytotoxicity against resistant MCF-7/Adr cells. These findings suggest that the mixed micelles have the potential to counteract MDR in clinical chemotherapy [151].

A study shows the ability of mixed micelles, which are coloaded with paclitaxel and curcumin and composed of Vitamin E and polyethylene glycol–phosphatidylethanolamine (PEG–PE), to overcome MDR in ovarian cancer [145]. Zhu et al. suggest codelivering Docetaxel and Vitamin E TPGS using PLGA nanoparticles. This uses TPGS's two functions: it can operate as an active-matrix component that inhibits P-gp ATPase to overcome MDR, and it can work as a pore-forming agent, resulting in smaller particles and faster drug release [152]. To combat MDR, reduction-sensitive DOX/KSV micelles demonstrated quick drug release in the tumour microenvironment, targeted solid tumours via the EPR effect and effectively disintegrated in the cytoplasm to release DOX and VES. As a result, P-gp expression was inhibited, ROS levels spiked, and tumour MDR was overcome synergistically, proving that these micelles were effective nanocarriers for the transport of anticancer drugs [153].

### 4.4. Vitamin K

Vitamin K exists in three main forms: Vitamin PK or phylloquinone (PK), Vitamin K2 or menaquinones (MKn) and Vitamin K3 or menadione [154]. Depending on the type, multiple sources of Vitamin K exists in multiple forms and can be obtained from various sources. PK is mostly present in green leafy vegetables (like kale), veggies belonging to the Brassica genus (like Brussels sprouts and broccoli), fruits (including avocado, kiwi and green grapes), herbs (like parsley and cilantro) and green and herbal beverages. Olive, canola and soybean oils are plant oils that are additional food sources [155–157]. They have a broad tissue distribution, have a protective role in the cardiovascular system, chronic kidney disease and bones [158, 159], and are engaged in a variety of biological processes, including cell differentiation, ectopic calcification, signal transduction, inflammation, proliferation, and bone homeostasis and blood coagulation [160–163]. Furthermore, several pathological illnesses, including dementia, some skin pathologies, RA, OA, osteoporosis, cancer, and functional decline and disability, have all been associated with Vitamin K insufficiency [155, 164].

Apart from the clotting mechanism, Vitamin K also has unique biochemical effects in regulating the cell cycle and has a beneficial effect on cancer treatment (Table 2). According to research, Vitamin K's role in influencing cellular processes such as apoptosis, cell cycle regulation and oxidative stress can complement chemotherapeutic approaches, especially in conditions where drug resistance is an issue. Research has shown that people who are receiving prolonged chemotherapies are more likely to be vitamin (including K) deficient [165, 166]. In such cases, supplementing Vitamin K can manage the deficiency as well as show promising effects in sensitising cancer cells to treatment, potentially enhancing the drug's efficacy [167].

According to a study by Dragh et al. on Drosophila, VK2 inhibits the generation of mtROS and functions as an electron carrier to prevent lymphoma while also repairing the structure and function of mitochondria [168]. A recent study shows that both the bortezomib (BTZ)-resistant and non-BTZ–resistant phenotypes of multiple myeloma cell growth were inhibited by supplementation with Vitamin D and Vitamin K, this indicates that the role of these vitamins in resistant cells [169]. Oh et al. identified menadione as a P-gp substrate, which is most likely the mechanism underlying the chemosensitising action. Apart from its inherent anticancer properties, menadione's capacity to evade drug resistance makes it a potentially useful chemotherapeutic enhancer [170].

Vitamin K analogues (K1, K2, K3 and K5) combined with chemotherapeutic medications is a safe and economical way to overcome drug resistance and improve chemotherapy results. Combination therapy with Vitamin K increases apoptosis, arrests the cell cycle and inhibits P-GP, which may increase the effectiveness of chemotherapy medications [171]. Shukla et al. revealed that the ABC drug transporter ABCG2, which is connected to MDR, has both Vitamin K3 and plumbagin as substrates. The ABCG2-mediated efflux of mitoxantrone was particularly blocked by plumbagin and Vitamin K3 [172]. A recent study by Nakaoka E et al. demonstrates that K3 and K5 are vitamin derivatives that partially inhibit the proliferation of MOLT-4 and MOLT-4/DNR cells by inducing apoptosis. They can also overcome medication resistance brought on by developing P-GP [173]. The synthetic vitamin K3 thio-derivative (VKT-1) inhibited tumour cell lines overexpressing ABC transporters (P-GP, ABCB5, BCRP) by causing cell arrest, inducing apoptosis, blocking migration and degrading microtubules in U2OS-GFP-α-tubulin cells [174]. In triple-negative breast cancer (TNCP), a study shows that Vitamin K2 augments the anticancer effect of 1,25 dihydroxy vitamin D3 (1,25(OH)2D3), promoting cell cycle arrest, differentiation and apoptosis through mechanisms that include increased VDR expression and do not rely on *γ*-carboxylation [175]. Also in OS cells, menadione and protocatechuic acid combined were more effective than either treatment alone, as seen by decreased cell viability and migration, decreased expression of NOX-2 and ROS and a modest increase in apoptosis [176]. All of those results were put forward as a theoretical assertion that adding Vitamin K to therapy regimens would improve outcomes for patients who are resistant to MTX.

## 5. MTX-ADRs and Mitigation by Vitamins

MTX causes multiple types of adverse drug reactions (ADRs) through distinct mechanisms. Hepatotoxicity is its primary toxicity. This occurs from ferritinophagy-mediated ferroptosis, where MTX causes ferritin to undergo autophagy, releasing free iron that can cause cell death and catalyse lipid peroxidation. High Mobility Group Box 1 (HMGB1)–mediated inflammation also exacerbates the situation [177].

MTX resistance and its relationship to nausea/vomiting involve complex interactions between pharmacological, neurochemical and behavioural pathways. Activation of serotonin (5-HT) receptors in the central nervous system and digestive is the main cause of these effects. Because of their increased sensitivity to the chemoreceptor trigger zone, adolescents are known to be six times more likely than adults to develop gastrointestinal intolerance [178]. Regarding the severity of nausea and vomiting in MTX resistance, there was a contradicting remark. According to a few studies, inadequate absorption into the cells and increased metabolism may result in less nausea and vomiting. According to other research, dose escalation as a compensatory measure may make the situation worse. Resistance can mitigate its toxic effects at the cellular level, but long-term use does not always result in the elimination of serotonergic activity as well as increased CTZ stimulation [178, 179].

Renal toxicity linked with MTX is mainly brought about by its accumulation due to insufficient renal elimination, resulting in elevated plasma concentrations that correspond with nephrotoxicity and an increased risk of infection. In turn, renal impairment prolongs exposure to elevated drug levels and exacerbates toxicity by further delaying MTX clearance. By modifying renal function and MTX elimination routes, conditions such as dehydration, low urine pH and some medicines might increase the risk of nephrotoxicity [180].

Haematologic toxicity is caused by folate deficiency due to DHFR inhibition, which impairs DNA synthesis and causes myelosuppression. Resistance mechanisms that require higher doses, like decreased drug absorption or increased TYMS expression, may make toxicity worse. This suppression can manifest as pancytopenia, leukopenia, neutropenia and megaloblastic anaemia, significantly increasing the risk of infections and mortality in severe cases [181].

When MTX builds up in brain tissues as a result of inadequate clearance and altered transport systems, it can negatively impact neuronal integrity and function and manifest as neurotoxicity or cognitive impairment. Potential dosage modifications are more likely to cause adverse effects in resistant patients undergoing HD-MTX therapy [182].

Fat-soluble vitamins may alleviate these ADRs [183, 184]:

1. Vitamin E neutralises lipid peroxidation, which prevents hepatic ferroptosis and could minimise MTX-induced hepatotoxicity.
2. Vitamin D enhances treatment efficacy in inflammatory conditions, allowing dose reduction, regulates immunological responses and improves renal calcium processing, potentially lowering the risk of nephrotoxicity. Also being a neuroprotective vitamin, it could minimise neural toxicity.
3. Vitamin A (although understudied in the available data) may aid in mucosal repair, addressing stomatitis or dermatologic consequences. Without affecting therapeutic efficacy, these vitamins may work in concert with MTX to improve tolerance.

## 6. Prospects and Research Focus

As personalised medicine advances, combining MTX with fat-soluble vitamins has great potential for improving therapeutic efficacy and counteracting MTX resistance via numerous mechanisms. Future studies should investigate the role of extrachromosomal DNA in resistance and the synergistic effects of such combinations on drug efflux mechanisms.

Furthermore, genomic and transcriptome profiling can discover biomarkers linked to MTX resistance, whereas pharmacogenomic research can modify treatment regimens based on individual genetic differences. Clinical trials and longitudinal studies may provide significant information about the safety and effectiveness of these combinations across a wide range of patient populations.

## 7. Conclusion

**Figure 2:**
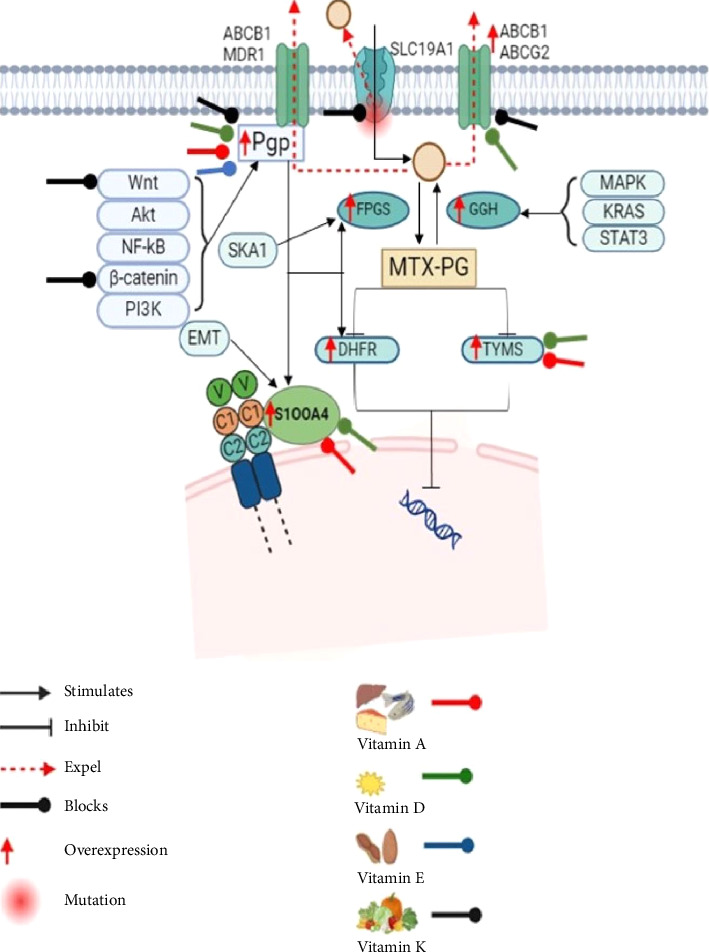
Schematic representation of the possible effects of fat-soluble vitamins in resistant pathways to overcome resistance. All fat-soluble vitamins could inhibit P-GP and prevent drug expulsion through efflux transporters. Furthermore, it acts on other efflux transporters, RFC, Wnt/β-catenin pathway, S100A4, DHFR and TYMS.

In conclusion, the combination of MTX with vitamins represents a promising strategy to mitigate the adverse events associated with MTX therapy while maximising therapeutic benefits (Figure 2). The diverse array of vitamins, particularly Vitamin A, Vitamin E and Vitamin D, offers potential avenues for tailored interventions to address specific MTX-related toxicities. Clinical evidence suggests that vitamin supplementation can effectively reduce multiple symptoms and improve overall tolerability without compromising the efficacy of existing anticancer medications (Tables 1 and 2). Moving forward, collaborative efforts between clinicians, researchers and pharmaceutical companies are crucial for advancing our understanding of combination therapy and translating these findings into clinical practice to optimise patient care.

## Figures and Tables

**Table 1:** Studies showing the effectiveness of Vitamin A in combination with chemotherapeutic drugs.

Vitamins	Drugs	Study type	Animal model	Observations
Vitamin A (10 μM)	Vinorelbine (0.1 μM)	In vitro and in vivo study	Engrafted female NOD/SCID mice were used to examine the breast cancer relapse.	• Potential to prevent the recurrence of breast cancer. • Used in conjunction with vinorelbine stealth liposome to treat and restrict the progression of breast cancer [93].

Vitamin A (6000 IU)	MTX	Clinical study	Not applicable	• Lowers β-HCG levels and accelerates trophoblastic cell regression. • Vitamin A coupled with MTX is more effective than MTX treating alone in eradicating cancer cells [94, 95].

Vitamin A as all-trans retinoic acid (ATRA) (25 mg/m^2^ to 45 mg/m^2^)	Daunorubicin and cytarabine	Clinical study	Not applicable	• Effective remission in acute promyelocytic leukaemia patients • Improved survival and low relapse rate [96]

**Table 2:** Studies showing the effectiveness of Vitamins D, E and K in combination with chemotherapeutic drugs.

Vitamins	Drugs	Study type	Animal model	Observations
Vitamin D (0.005%)	5-fluorouracil (5%)	In vivo and clinical study	Genetically engineered WT animals were used to evaluate the mechanism of calcipotriol against skin carcinogenesis.	• 5-fluorouracil and calcipotriol work together to efficiently initiate CD4+ T cell–mediated immunity • Protects against actinic keratoses as well as maybe other cancers of the skin and organs [143].

Vitamin E (15 mg/kg)	Doxorubicin (5 mg/kg)	In vivo study	BALB/c nude mice xenograft tumour model was used to study the antitumour effect of treatment.	• Vitamin E succinate and doxorubicin-loaded nano vesicles show effective antitumour effect. • Reverse drug resistance in drug-resistant human chronic myelogenous leukaemia [144].

Vitamin E micelle–loaded drug (5 mM)	Paclitaxel (10 mg/kg)	In vitro and in vivo study	Female nude mice bearing SKOV-3 sensitive and resistant tumours to study the tumour inhibition.	• The formulation successfully reversed the resistant human ovarian cancer. • It shows considerable improvement in paclitaxel effectiveness [145].

Vitamin E succinate (150 mg/kg)	MTX (180 mg/kg)	In vivo study	Female BALB/c mice were used to evaluate the combinational therapy.	• Enhance high-dose MTX efficacy in the 4T1 breast tumour model [146].

Vitamin K (15 mg)	Sorafenib (400 mg)	Clinical study	Not applicable	• Improve the treatment results for patients with hepatocellular carcinoma [147].

## Data Availability

Data sharing is not applicable to this article as no datasets were generated or analysed during the current study.
